# Anti‐oligomeric PRI‐002 does not cause ARIA‐E in an ongoing Phase 2 trial

**DOI:** 10.1002/alz70861_108724

**Published:** 2025-12-23

**Authors:** Oliver Peters, Dagmar Jürgens, Gerhard Tischler, Alexander Brener, Tina Bartsch, Gunther Kauselmann, Knut Adermann, Kathrin Zeiger, Katja Lindner, Julie‐Anne Gabelich, Dieter Willbold

**Affiliations:** ^1^ Charité – Universitätsmedizin Berlin, Berlin Germany; ^2^ German Center for Neurodegenerative Diseases (DZNE), Berlin Germany; ^3^ Priavoid, Düsseldorf, NRW Germany; ^4^ PRInnovation, Düsseldorf, NRW Germany; ^5^ Forschungszentrum Julich, Julich, Priavoid GmbH Germany; ^6^ Heinrich‐Heine‐Universität Düsseldorf, Düsseldorf, NRW Germany

## Abstract

**Background:**

Amyloid related imaging abnormalities (ARIA) are a common side effect of monoclonal anti‐amyloid antibodies and limit the benefit risk ratio. Here we investigate the safety and efficacy of the anti‐oligomeric all‐D‐peptide PRI‐002 in early AD. PRI‐002 is an orally available, first in class compound developed to disassemble neurotoxic Aβ oligomers into non‐toxic Aβ monomers. In a phase 1 B study PRI‐002 has been shown to improve learning and memory function in early AD patients after 4 weeks of once‐daily oral treatment and 4 weeks follow‐up (Kutzsche et al., Nat. Commun. 2025).

**Method:**

PRImus‐AD (NCT06182085) is an ongoing randomized, double‐blind, placebo‐controlled phase 2 study of PRI‐002 in early symptomatic AD receiving either 300 mg, 600 mg or placebo every day for at least 48 weeks. Safety is measured by comparing adverse and serious adverse events in verum groups versus placebo. Primary efficacy outcome is CDR‐SB. According to study protocol intense MRI monitoring to detect possible ARIA‐E were performed during titration.

**Result:**

304 patients 70.4±6.5 years on age, with a MMSE of 25.2±2.2, 155 with MCI (51.0%) and 149 (49.0%) with mild dementia were randomized. CDR‐SB at baseline was 3.3±1.4 and RBANS‐DMI 54.8±12.7. 208 patients have at least one APOE4 allele (68.4%). After 90 patients, irrespective of the treatment arm, had passed the first 24 weeks of treatment, the incidence of ARIA was evaluated. Within these 90 patients ARIA‐E occurred in 4 (4.4%) and ARIA‐H were detected in 13 (14.4%) subjects. This incidence is in line with large observational cohorts like ADNI. An unblinded DSMB reviewed the ARIA incidence in all three study arms and recommended to stop the intense ARIA monitoring for the rest of the study, since no imbalance was detected.

**Conclusion:**

In an ongoing phase 2 trial with oral PRI‐002 that was designed to disassemble synaptotoxic Aβ aggregates into harmless Aβ monomers, the study drug is safe and in detail does not cause ARIA incidents Efficacy data are expected in 2026.

Kutzsche J et al. Oral PRI‐002 treatment in patients with MCI or mild AD. Nat. Commun. *accepted*, (2025).

